# Global Existence and Energy Decay Rates for a Kirchhoff-Type Wave Equation with Nonlinear Dissipation

**DOI:** 10.1155/2014/716740

**Published:** 2014-04-07

**Authors:** Daewook Kim, Dojin Kim, Keum-Shik Hong, Il Hyo Jung

**Affiliations:** ^1^Department of Mathematics, Pusan National University, 30 Jangjeon-dong, Geumjeong-gu, Busan 609-735, Republic of Korea; ^2^Department of Mathematics, Oregon State University, Corvallis, OR 97331, USA; ^3^Department of Cogno-Mechatronics Engineering and School of Mechanical Engineering, Pusan National University, Busan 609-735, Republic of Korea

## Abstract

The first objective of this paper is to prove the existence and uniqueness of global solutions for a Kirchhoff-type wave equation with nonlinear dissipation of the form *Ku*′′ + *M*(|*A* ^1/2^ *u*|^2^)*Au* + *g*(*u*′) = 0 under suitable assumptions on *K*, *A*, *M*(·), and *g*(·). Next, we derive decay estimates of the energy under some growth conditions on the nonlinear dissipation *g*. Lastly, numerical simulations in order to verify the analytical results are given.

## 1. Introduction

A mathematical model for the transverse deflection of an elastic string of length *L* > 0 whose ends are held a fixed distance apart is written in the form of the hyperbolic equation

(1) $$\begin{array}{c} \frac{\partial^{2}u\left(x,t\right)}{\partial t^{2}}-\left(\alpha +\beta \overset{L}{\underset{0}{\int }}{\left|\frac{\partial u\left(x,t\right)}{\partial x}\right|}^{2}dx\right)\frac{\partial^{2}u\left(x,t\right)}{\partial x^{2}}=0, \end{array}$$

which was proposed by Kirchhoff [1], where *u*(*x*, *t*) is the deflection of the point *x* of the string at the time *t* and *α* > 0, *β* are constants. Kirchhoff first introduced (1) in the study of the oscillations of stretched strings and plates, so that (1) is called the wave equation of Kirchhoff type. The Kirchhoff-type model also appeared in scientific research for beam or plate [2–5]. Such nonlinear Kirchhoff model gives one way to describe the dynamics of an axially moving string. In recent years, axially moving string-like continua such as wires, belts, chains, and band saws have been the subject of study of researchers [6–14].

The mathematical aspects of the natural generalization of the model (1) in *Ω* ⊂ ℝ^*n*^:

(2) $$\begin{array}{c} u^{\prime \prime }-M\left(\int_{\Omega }{\left|\nabla u\right|}^{2}dx\right)\Delta u+g\left(u^{\prime }\right)=0, \end{array}$$

(3) $$\begin{array}{c} u\left(0\right)=u_{0},\;\;u^{\prime }\left(0\right)=u_{1}, \end{array}$$

under some assumptions on *M*(·), *g*(·), have been studied, using different methods, by many authors [6, 8, 15–22].

When *g*(·) = 0 and *n* = 1, the problem (2)-(3) was studied by Dickey [16] and Bernstein [15] who considered analytic functions as the initial data (see also Yamada [21] and Ebihara et al. [17]). In case when *g*(·) = 0 and *n* ≥ 1, Pohožaev [22] obtained the existence and uniqueness of global solutions for the problem (2)-(3). Lions [20] also formulated Pohožaev's results in an abstract context and obtained better results.

Equation (2) with linear dissipative term, that is, *g*(*u*′) = *δu*′  (*δ* > 0), was investigated by Mizumachi [23], Nishihara and Yamada [24], Park et al. [25], and Jung and Choi [26]. In fact, they studied the existence, uniqueness, and the energy decay rates of solutions for the problem (2)-(3). On the other hand, related works to a Kirchhoff-type equation with *Ku*′′ instead of *u*′′ can be found in Levine [19]. Jung and Lee [27] got the result for a Kirchhoff-type equation with strong dissipative term. But they studied a simple form with the coefficient *M*(·) ≡ 1. In case of the equation concerning nonlinear Kirchhoff-type coefficient, recently, Kim et al. [8], Ghisi and Gobbino [28], and Aassila and Kaya [29] have studied existence and energy decay rates of global (or local) solutions for the equation. By giving some suitable smallness conditions on the sizes of the initial data, they assured global existence and energy decay rates for the solutions.

In this paper, we study the existence, uniqueness, and the decay estimates of the energy for a class of Kirchhoff-type wave equations in a Hilbert space *H*:

(4) $$\begin{array}{c} Ku^{\prime \prime }+M\left({\left|A^{1/2}u\right|}^{2}\right)Au+g\left(u^{\prime }\right)=0\;\text{in}\;H,\\ u\left(0\right)=u_{0},\;\left(Ku^{\prime }\right)\left(0\right)=K^{1/2}u_{1}, \end{array}$$

where *K* and *A* are linear operators in *H* and *M*(·) ∈ *C* ^1^[0, *∞*). For global existence of this problem, we give some suitable smallness conditions. So, the main contribution of these results is to consider a general model which contains the concrete model (2)-(3) and to improve the results of Kouémou-Patcheu [30] and Jung and Choi [26]. Moreover, as an application, we give some simulation results about solution's shapes and the algebraic decay rate for a Kirchhoff-type wave equation with nonlinear dissipation.

The method applied in this paper is based on the multipliers technique [31], Galerkin's approximate method, and some integral inequalities due to Haraux [32].

This paper is organized as follows. In Section 2, we recall the notation, hypotheses, and some necessary preliminaries and prove the existence and uniqueness of global solutions for the system (4) by employing Feado-Galerkin's techniques under suitable smallness condition. In Section 3, we derive the energy decay rates by using the multiplier technique under suitable growth conditions on *g*. Finally, in Section 4, we give an example and its numerical simulations to illustrate our results.

## 2. Preliminaries and Existence

Let *Ω* be a bounded open domain in ℝ^*n*^ having a smooth boundary Γ and *H* = *L* ^2^(*Ω*) with inner product and norm denoted by (·, ·) and |·|, respectively. Let *K* be a linear, positive, and self-adjoint operator on *H*; that is, there is a constant *c* > 0 such that

(5) $$\begin{array}{c} \left(Ku,u\right)\geq c{\left|u\right|}^{2},\;\forall u\in H. \end{array}$$

Let *A* be a linear, self-adjoint, and positive operator in *H*, with domain *V* : = *D*(*A*) dense in *H*, *KA* = *AK* on *D*(*A*)∩*D*(*K*), and the graph norm denoted by ||·||. We assume that the imbedding *V* ⊂ *H* is compact. Identifying *H* and its dual *H*′, it follows that *V* ⊂ *H* ⊂ *V*′, where *V*′ is the dual of *V*. Let 〈·, ·〉_*V*′,*V*_ denote the duality pairing between *V*′ and *V* and *W* : = *D*(*A* ^1/2^).

Throughout the paper we will make the following assumptions.

(M) *M*(*s*) is a *C* ^1^[0, *∞*) real function and *M*′(*s*) ≥ 0. Furthermore, there exist some positive constants *β* and *γ*_0_ such that *M*(*s*) ≥ *β* > 0 for all *s* ≥ 0 and |*M*′(*s*)*s* | /*M*(*s*) ≤ *γ*_0_.
(G) *g* : ℝ → ℝ is a nondecreasing continuous function such that *g*(0) = 0 and there is a constant *k* > 0 and *q* ≥ 1 such that
  (6) $$\begin{array}{c} \left|g\left(x\right)\right|\leq k\left(1+{\left|x\right|}^{q}\right)\;\forall x\in \mathbb{R}. \end{array}$$
  And (*g*(*u*), *Au*) ≥ 0 for all *u* ∈ *D*(*A*)∩*D*(*A* ^1/2^). Note that the last assumption of (G) makes sense. In fact, when *A* = −Δ and *g*(*u*) = |*u*|^*α*^ *u*, *α* ≥ 1, we can easily show that (*g*(*u*), *Au*) ≥ 0 for all *u* ∈ *D*(*A*)∩*D*(*A* ^1/2^).
(H) *M*′(*s*) > *M*(*s*)|*g*(*x*)|, *s* ∈ [0, *∞*), *x* ∈ ℝ.
(S) *V* ⊂ *L* ^*q*+1^(*Ω*) for some *q* ≥ 1.

Let $\bar{M}(t)$ and *E*(*t*) be defined as follows:

(7) $$\begin{array}{c} \bar{M}\left(t\right)=\overset{t}{\underset{0}{\int }}M\left(s\right)ds \end{array}$$

(8) $$\begin{array}{c} E\left(t\right)=\frac{1}{2}\left[{\left|K^{1/2}u^{\prime }\right|}^{2}+\bar{M}\left({\left|A^{1/2}u\right|}^{2}\right)\right]. \end{array}$$

And also let us consider the functions

(9) P(t):=|K1/2u′(t)|2M(|A1/2u|2)+|A1/2u(t)|2,Q(t):=|K1/2A1/2u′(t)|2M(|A1/2u|2)+|Au(t)|2,G(t):=|K1/2u′(t)|M(|A1/2u|2).

**Theorem 1**

Let the initial conditions (*u*_0_, *u*_1_) ∈ *W* × *L* ^2*q*^(*Ω*) satisfy the smallness assumption

(10) $$\begin{array}{c} {||M^{\prime }||}_{L^{\infty }([0,P(0)])}B\left(u_{0},u_{1}\right)\sqrt{Q\left(0\right)}<\frac{1}{4}, \end{array}$$

where *B*(*u*_0_, *u*_1_) = max⁡⁡{|*K* ^1/2^ *u*_1_ | /*M*(|*A* ^1/2^ *u*_0_|^2^), *M*(|*A* ^1/2^ *u*_0_|^2^)/((*M*(|*A* ^1/2^ *u*_0_|^2^))′  −  $g(u_{1})M(|A^{1/2}u_{0}{|}^{2})))\sqrt{Q(0)}\}$. Then there is a unique function *u* ∈ *L* ^*∞*^(0, *T*; *W*)∩*W* ^1,*∞*^(0, *T*; *V*)∩*W* ^2,*∞*^(0, *T*; *H*) such that, for any *T* > 0,

(11) Ku′′+M(|A1/2u|2)Au+g(u′)=0in  L(q+1)/q(0,T;V′),

(12) $$\begin{array}{c} u\left(0\right)=u_{0},\;\;\left(Ku^{\prime }\right)\left(0\right)=K^{1/2}u_{1}. \end{array}$$

**Proof**

Assume that, for simplicity, *V* is separable; then there is a sequence (*e* ^*j*^)_*j*≥1_ consisting of eigenfunctions of the operator *A* corresponding to positive real eigenvalues *μ*_*j*_ tending to +*∞* so that *Ae* ^*j*^ = *μ*_*j*_ *e* ^*j*^, *j* ≥ 1.

Let us denote by *V*_*m*_ the linear hull of *e* ^1^, *e* ^2^,…, *e* ^*m*^. Note that (*e* ^*j*^)_*j*≥1_ is a basis of *H*, *V*, and *W* and hence it is dense in *H*, *V*, and *W*.

*Approximate Solutions*. We search for a function *u*_*m*_(*t*) = ∑_*j*=1_^*m*^ *g*_*jm*_(*t*)*e* ^*j*^ such that, for any *v* ∈ *V*_*m*_, *u*_*m*_(*t*) satisfies the approximate equation

(13) $$\begin{array}{c} \left(Ku_{m}^{\prime \prime }\left(t\right)+M\left({\left|A^{1/2}u_{m}\right|}^{2}\right)Au_{m}+g\left(u_{m}^{\prime }\right),v\right)=0 \end{array}$$

and the initial conditions as the projections of *u*_0_ and *u*_1_ over *V*_*m*_ satisfy

(14) $$\begin{array}{c} u_{m}\left(0\right)=u_{0m}=\overset{m}{\underset{j=1}{\sum }}\left(u_{0},e^{j}\right)e^{j}⟶u_{0}\;\text{in}\;W \end{array}$$

(15) (Kum′)(0)=K1/2u1m=∑j=1m(u1,ej)ej⟶K1/2u1 in  L2q(Ω).

For *v* = *e* ^*j*^, *j* = 1,2,…*m*, the system (13)–(15) of ordinary differential equations of variable *t* has a solution *u*_*m*_(*t*) in an interval [0, *t*_*m*_).

Now we obtain a priori estimates for the solution *u*_*m*_(*t*) and it can also be extended to [0, *T*) for all *T* > 0.

*A Priori Estimate I*. Let us consider *v* = *u*_*m*_′ in (13). Using (7), we have

(16) ddt(|K1/2um′(t)|2+M¯(|A1/2um(t)|2)) +2(g(um′(t)),um′(t))=0.

Integrating (16) over (0, *t*), *t* ≤ *t*_*m*_, and using (8), we have

(17) 2E(0)=[|K1/2um′(t)|2+M¯(|A1/2um(t)|2)]+2∫0t(g(um′(s)),um′(s))ds.

Using (5) and (7), we deduce that

(18) 2E(0)≥|K1/2um′(t)|2+β|A1/2um(t)|2+2∫0t∫Ωum′(s)g(um′(s))dx ds,

where the left-hand side is constant independent of *m* and *t*. Thus estimation (18) yields, for any 0 < *T* < *∞*,

(19) $$\begin{array}{c} u_{m}^{\prime }\;\text{bounded}\;\text{in}\;L^{\infty }\left(0,T;H\right), \end{array}$$

(20) $$\begin{array}{c} K^{1/2}u_{m}^{\prime }\;\;\text{bounded}\;\;\text{in}\;L^{\infty }\left(0,T;H\right), \end{array}$$

(21) $$\begin{array}{c} A^{1/2}u_{m}\;\text{bounded}\;\text{in}\;L^{\infty }\left(0,T;H\right), \end{array}$$

(22) $$\begin{array}{c} u_{m}^{\prime }g\left(u_{m}^{\prime }\right)\;\text{bounded}\;\text{in}\;L^{1}\left(\left[0,T\right]\times \Omega \right). \end{array}$$

Now we show that *u*_*m*_(*t*) can be extended to [0, *∞*). We need the following smallness assumption:

(23) ||M′||L∞([0,P(0)]) ×max⁡{|K1/2um1|M(|A1/2um0|2),(M(|A1/2um0|2))×((M(|A1/2um0|2))′−g(um1)×M(|A1/2um0|2))−1Q(0)} ×Q(0)<14,

where *P*(0) = (|*K* ^1/2^ *u*_*m*1_|^2^/*M*(|*A* ^1/2^ *u*_*m*0_|^2^)) + |*A* ^1/2^ *u*_*m*0_|^2^, *Q*(0) = (|*K* ^1/2^ *A* ^1/2^ *u*_*m*1_|^2^  /  *M*(|*A* ^1/2^ *u*_*m*0_|^2^)) + |*Au*_*m*0_|^2^.

Let [0, T*) be the maximal interval where the solution exists. Set *Z*(*t*): = *M*(|*A* ^1/2^ *u*_*m*_(*t*)|^2^) and

(24) $$\begin{array}{c} T:=\sup\left\{\tau \in \left[0,T^{*}\right)\;|\;\left|\frac{Z^{\prime }\left(t\right)}{Z\left(t\right)}\right|\leq \frac{1}{2},\;\;Z\left(t\right)>0,\;\;\forall t\in \left[0,\tau \right)\right\}. \end{array}$$

With simple computations it follows that

(25) P′(t)=−1Z(t)(2(g(um′(t)),um′(t))+Z′(t)Z(t)|um′(t)|2)  ≤0,

(26) Q′(t) =−1Z(t)(2(g(um′(t)),Aum′(t))+Z′(t)Z(t)|A1/2um′(t)|2) ≤0,

(27) (G2)′(t) ≤−G(t){2(Z′(t)Z(t)−|g(um′(t))|)G(t)−2|Aum(t)|},

for all *t* ∈ [0, *T*).

Next, we show that *T* = T*. Let us assume by contradiction that *T* < T*. Since |*Z*′(*t*)|≤(1/2)*Z*(*t*) in [0, *T*), we have that

(28) $$\begin{array}{c} 0<Z\left(0\right)e^{-T/2}\leq Z\left(T\right)\leq Z\left(0\right)e^{T/2}. \end{array}$$

Since *Z*(*t*) and *Z*′(*t*) are continuous functions, by the maximality of *T* we have that necessarily

(29) $$\begin{array}{c} \left|\frac{Z^{\prime }\left(t\right)}{Z\left(t\right)}\right|=\frac{1}{2}. \end{array}$$

From (88) and (89) it follows that *P* and *Q* are nonincreasing functions; hence

(30) $$\begin{array}{c} {\left|A^{1/2}u_{m}\left(t\right)\right|}^{2}\leq P\left(t\right)\leq P\left(0\right),\\ {\left|Au_{m}\left(t\right)\right|}^{2}\leq Q\left(t\right)\leq Q\left(0\right). \end{array}$$

Moreover by Lemma 3.1 in [28] we have that

(31) G(t)≤max⁡{G(0),Z(0)Z′(0)−g(um1)Z(0)Q(0)},  ∀t∈[0,T].

By (91)–(31), and the smallness assumption (23), we have that

(32) |Z′(T)Z(T)|=|2M′(|A1/2um(t)|2)(um′(T),Aum(T))Z(T)|≤2max⁡0≤r≤P(0)|M′(r)||um′(T)|Z(T)|Aum(T)|≤2max⁡0≤r≤P(0)|M′(r)|×max⁡{G(0),Z(0)Z′(0)−g(um1)Z(0)Q(0)}×Q(0)<12.

This contradicts (29). Therefore it follows that *u*_*m*_(*t*) can be extended to [0, *T*) for any *T* ∈ (0, *∞*).

Furthermore, putting *v* = *Au*_*m*_′ in (13), we get

(33) $$\begin{array}{c} \frac{\left(Ku_{m}^{\prime \prime },Au_{m}^{\prime }\right)}{M\left({\left|A^{1/2}u_{m}\right|}^{2}\right)}+\left(Au_{m},Au_{m}^{\prime }\right)+\frac{\left(g\left(u_{m}^{\prime }\right),Au_{m}^{\prime }\right)}{M\left({\left|A^{1/2}u_{m}\right|}^{2}\right)}=0. \end{array}$$

From this we obtain

(34) 12ddt((Kum′,Aum′)M(|A1/2um|2)+|Aum|2)+(g(um′),Aum′)M(|A1/2um|2) =−(Kum′,Aum′)M′(|A1/2um|2)(A1/2um′,A1/2um){M(|A1/2um|2)}2.

Integrating (34) over (0, *t*) and taking into account assumptions (M) and (G), and applying Gronwall's inequality, we obtain

(35) $$\begin{array}{c} Au_{m}\;\text{bounded}\;\text{in}\;L^{\infty }\left(0,T;H\right). \end{array}$$

From (6) and (22), it follows that

(36) $$\begin{array}{c} g\left(u_{m}^{\prime }\right)\;\text{bounded}\;\text{in}\;L^{(q+1)/q}\left(\left[0,T\right]\times \Omega \right). \end{array}$$

*A Priori Estimate II*. Taking *v* = *u*_*m*_′′(*t*) in (13) and choosing *t* = 0, we obtain

(37) |K1/2um′′(0)|2+(M(|A1/2u0m|2)Au0m+g(u1m),um′′(0)) =0.

Thus we have

(38) |K1/2um′′(0)|2≤(|g(u1m)|+|M(|A1/2u0m|2)Au0m|)|um′′(0)|≤(|g(u1m)|+|M(|A1/2u0|2)Au0|)×|K1/2um′′(0)|.

Thanks to the assumption (6), we deduce from (15) that

(39) $$\begin{array}{c} \left(g\left(u_{1m}\right)\right)\;\;\text{is}\;\text{bounded}\;\text{in}\;L^{2}\left(\Omega \right). \end{array}$$

Therefore we conclude that the right-hand side is bounded; that is,

(40) $$\begin{array}{c} K^{1/2}u_{m}^{\prime \prime }\left(0\right)\;\;\text{bounded}\;\text{in}\;H. \end{array}$$

*A Priori Estimate III*. For *t* < *T*, we apply (13) at points *t* and *t* + *ζ* such that 0 < *ζ* < *T* − *t*. By taking the difference *v* = *u*_*m*_′(*t* + *ζ*) − *u*_*m*_′(*t*) in (13) and the assumption (G), we obtain

(41) 0≥(Kum′′(t+ζ)−Kum′′(t),um′(t+ζ)−um′(t))+(M(|A1/2um(t+ζ)|2)Aum(t+ζ)  −M(|A1/2um(t)|2)Aum(t),um′(t+ζ)−um′(t)).

Thus we have

(42) 0≥12ddt[|K1/2(um′(t+ζ)−um′(t))|2]+M(|A1/2um(t+ζ)|2)×(Aum(t+ζ)−Aum(t),um′(t+ζ)−um′(t))+[M(|A1/2um(t+ζ)|2)−M(|A1/2um(t)|2)]×(Aum(t),um′(t+ζ)−um′(t)).

Set

(43) $$\begin{array}{c} {𝚽}_{\zeta m}\left(t\right)={\left|K^{1/2}\left(u_{m}^{\prime }\left(t+\zeta \right)-u_{m}^{\prime }\left(t\right)\right)\right|}^{2}. \end{array}$$

By using (42), Young's inequality, the assumption (M), and the fact that *K* is positive self-adjoint operator, we see that *𝚽*_*ζm*_′(*t*) ≤ *c𝚽*_*ζm*_(*t*). Therefore we deduce

(44) $$\begin{array}{c} {𝚽}_{\zeta m}\left(t\right)\leq {𝚽}_{\zeta m}\left(0\right)\exp\left(cT\right)\;\forall t\in \left[0,T\right]. \end{array}$$

Dividing the two sides of (44) by *ζ* ^2^, letting *ζ* → 0, and using (43), we deduce

(45) $$\begin{array}{c} c{\left|u_{m}^{\prime \prime }\right|}^{2}\leq {\left|K^{1/2}u_{m}^{\prime \prime }\left(0\right)\right|}^{2}. \end{array}$$

From (40), it follows that |*u*_*m*_′′|^2^ ≤ *C*.

Since *u*_*m*_ ∈ *C* ^2^[0, *T*], the previous inequality is verified for all *t* ∈ [0, *T*]. Therefore we conclude that

(46) $$\begin{array}{c} u_{m}^{\prime \prime }\;\text{bounded}\;\text{in}\;L^{\infty }\left(0,T;H\right). \end{array}$$

Moreover, using (19) and (46), it follows that

(47) $$\begin{array}{c} u_{m}^{\prime }\;\text{bounded}\;\text{in}\;L^{2}\left(0,T;H\right),\\ u_{m}^{\prime \prime }\;\text{bounded}\;\text{in}\;L^{2}\left(0,T;H\right). \end{array}$$

Applying a compactness theorem given in [33], we obtain

(48) $$\begin{array}{c} u_{m}^{\prime }\;\text{precompact}\;\text{in}\;L^{2}\left(0,T;H\right). \end{array}$$

*Passage to the Limit*. Applying the Dunford-Pettis theorem, we conclude from (19), (21), (36), and (46)-(48), replacing the sequence *u*_*m*_ with a subsequence if needed, that

(49) $$\begin{array}{c} u_{m}⟶u\;\text{weak-star}\;\text{in}\;L^{\infty }\left(0,T;V\right), \end{array}$$

(50) $$\begin{array}{c} u_{m}^{\prime }\;⟶u_{}^{\prime }\;\text{weak-star}\;\text{in}\;L^{\infty }\left(0,T;H\right), \end{array}$$

(51) $$\begin{array}{c} u_{m}^{\prime \prime }⟶u_{}^{\prime \prime }\;\text{weak-star}\;\text{in}\;L^{\infty }\left(0,T;H\right), \end{array}$$

(52) $$\begin{array}{c} u_{m}^{\prime }\;⟶u_{}^{\prime }\;\text{a}.\text{e}\;\text{in}\;\Omega \times \left[0,T\right], \end{array}$$

(53) $$\begin{array}{c} g\left(u_{m}^{\prime }\;\right)⟶\psi \;\;\text{weak-star}\text{in}\;L^{(q+1)/q}\left(0,T;H\right), \end{array}$$

(54) $$\begin{array}{c} M\left({\left|A^{1/2}u_{m}\right|}^{2}\right)Au_{m}⟶\chi \;\text{weak-star}\;\text{in}\;L^{\infty }\left(0,T;H\right) \end{array}$$

for suitable functions *u* ∈ *L* ^*∞*^(0, *T*; *V*), *χ* ∈ *L* ^*∞*^(0, *T*; *H*), and *ψ* ∈ *L* ^(*q*+1)/*q*^(**Ω** × [0, *T*]).

Now we are going to show that *u* is a solution of the problem (11)-(12). Indeed, from (49) to (51), we have

(55) $$\begin{array}{c} \int_{\Omega }u_{m}\left(0\right)e^{j}dx⟶\int_{\Omega }u\left(0\right)e^{j}dx,\;\\ \int_{\Omega }u_{m}^{\prime }\left(0\right)e^{j}dx⟶\int_{\Omega }u^{\prime }\left(0\right)e^{j}dx \end{array}$$

for each fixed *j* ≥ 1. So we conclude that, for any *j* ≥ 1,

(56) ∫Ω(um(0)−u0)ejdx=∫Ω(u′(0)−u1)ejdx  =  0as  m⟶∞,

which shows that (12) holds.

We will prove that, in fact, *χ* = *M*(|*A* ^1/2^ *u*|^2^)*Au*; that is,

(57) M(|A1/2um|2)Aum⟶M(|A1/2u|2)Auweak-star  in  L∞(0,∞;H).

For *v* ∈ *L* ^2^(0, *T*; *H*), we have

(58) ∫0T(χ−M(|A1/2u|2)Au,v)dt =∫0T(χ−M(|A1/2um|2)Aum,v)dt  +∫0TM(|A1/2u|2)(Aum−Au,v)dt  +∫0T(M(|A1/2um|2)−M(|A1/2u|2))    ×(Aum,v)dt.

We deduce from (49) and (54) that the first and second terms in (58) tend to zero as *m* → *∞*. For the last term, using the fact that *M* is *C* ^1^ and (21), we can derive (with some positive constants *c*_1_, *c*_2_)

(59) ∫0T(M(|A1/2um|2)−M(|A1/2u|2))(Aum,v)dt ≤c1∫0T|A(um+u),um−u|dt ≤c2(∫0T|um−u|2dt)1/2.

Since *u*_*m*_ is bounded in *L* ^*∞*^(0, *T*; *V*) and the injection of *V* in *H* is compact, we have

(60) $$\begin{array}{c} u_{m}⟶u\;\text{strongly}\;\text{in}\;L^{2}\left(0,T;H\right). \end{array}$$

From (58) to (60), we deduce (57). It follows from (49), (51), and (57) that, for each fixed *v* ∈ *L* ^*q*+1^(0, *T*; *V*),

(61) ∫0T(Kum′′+M(|A1/2um|2)Aum,v)dt ⟶∫0T(Ku′′+M(|A1/2u|2)Au,v)dt

as *m* → +*∞*.

For the nonlinear term, *g*(*u*′), it remains to show that, for any fixed *v* ∈ *L* ^*q*+1^(0, *T*; *V*),

(62) $$\begin{array}{c} \overset{T}{\underset{0}{\int }}\int_{\Omega }vg\left(u_{m}^{\prime }\right)dx\;dt⟶\overset{T}{\underset{0}{\int }}\int_{\Omega }vg\left(u^{\prime }\right)dx\;dt \end{array}$$

as *m* → *∞*.

At this moment we use the following lemma due to Jung and Choi (see [26, page 12]).

**Lemma 2**

Suppose that *Ω* × [0, *T*] is a bounded open domain of ℝ^*n*^ × ℝ; *g*_*m*_ and *g* are in *L* ^*q*^(*Ω* × [0, *T*]), 1 < *q* < *∞*, such that *g*_*m*_ → *g* a.e., in *Ω* × [0, *T*]. Then *g*_*m*_ → *g* weakly in *L* ^*q*^(*Ω* × [0, *T*]).

From (53), *g*(*u*_*m*_′) → *g*(*u*′) a.e. in *Ω* × [0, *T*]. By (36), we can use the above lemma and so we have *ψ* = *g*(*u*′); that is,

(63) $$\begin{array}{c} g\left(u_{m}\right)⟶g\left(u\right)\;\text{weak}\;\text{in}\;L^{(q+1)/q}\left(\Omega \times \left(0,T\right)\right), \end{array}$$

which implies (62). Therefore we obtain

(64) ∫0T(Ku′′+M(|A1/2u|2)Au+g(u′),v)dt=0,∀v∈Lq+1(0,T;V).

The uniqueness is obtained by a standard method, so we omit the proof here.

## 3. Energy Estimates

In this section we study the energy estimate under suitable growth conditions on *g*.

Let us assume that there exist a number *p* ≥ 1 and positive constants *c*_1_, *i* = 1,2, such that

(65) c1min⁡{|K1/2x|,|K1/2x|p} ≤|g(x)|≤c2max⁡{|K1/2x|,|K1/2x|1/p}

for all *x* ∈ ℝ.

**Theorem 3**

Assume that (65) holds. Then one obtains the following energy decay:

(66) $$\begin{array}{c} E\left(t\right)\leq \{\begin{array}{cc} c_{0}E\left(0\right)e^{-wt}\;\;\forall t\geq 0, & if\;\;p=1,\\ {\tilde{c}}_{0}{\left(1+t\right)}^{-2/(p-1)}\;\;\forall t\geq 0, & if\;\;p>1, \end{array} \end{array}$$

where *c*_0_, *w*, and ${\tilde{c}}_{0}$ are some positive constants.

**Proof**

Let *T* > 0 be arbitrary and fixed and let *u* ∈ *L* ^*∞*^(0, *T*; *V*)∩*W* ^2,*∞*^(0, *T*; *H*) be a solution of (11) and (12). Multiplying (11) by *u*′ and integrating by parts in *Ω* × (*s*, *T*)  (0 ≤ *s* < *T*), we obtain that

(67) $$\begin{array}{c} E\left(T\right)-E\left(s\right)=-\overset{T}{\underset{s}{\int }}\left(g\left(u^{\prime }\left(t\right)\right),u^{\prime }\left(t\right)\right)dt. \end{array}$$

By (*g*(*u*′(*t*)), *u*′(*t*)) ≥ 0 and being the primitive of an integrable function, it follows that the energy *E* is nonincreasing, locally absolutely continuous and

(68) $$\begin{array}{c} E^{\prime }\left(t\right)=-\left(g\left(u^{\prime }\left(t\right)\right),u^{\prime }\left(t\right)\right)\;\text{a}.\text{e}.\;\text{in}\;\left[0,\infty \right). \end{array}$$

Here and in what follows we will denote by *c* diverse positive constants. We are going to show that the energy of this solution satisfies

(69) $$\begin{array}{c} \overset{T}{\underset{s}{\int }}E{\left(t\right)}^{(p+1)/2}\leq cE\left(s\right)\;\forall 0\leq s\leq T<\infty . \end{array}$$

Once (69) is satisfied, the integral inequalities given in Komornik [31] and Haraux [32] will establish (66).

Now, multiplying (11) by *E*(*t*)^(*p*−1)/2^ *u* and integrating by parts, we have

(70) 0=∫sTE(t)(p−1)/2(Ku′′+M(|A1/2u|2)Au+g(u′),u)dt=[E(t)(p−1)/2(Ku′,u)]sT−p−12∫sTE(t)(p−3)/2E′(t)(Ku′,u)dt−∫sTE(t)(p−1)/2|K1/2u′|2dt+∫sTE(t)(p−1)/2(M(|A1/2u|2)|A1/2u|2,u)dt+∫sTE(t)(p−1)/2(g(u′),u)dt.

Note that by the assumption (M) and (21), we can choose some positive number

(71) $$\begin{array}{c} \alpha =\underset{s\in [0,{\left|A^{1/2}u\right|}^{2}]}{\max}\left\{M\left(s\right)\right\}<\infty \end{array}$$

so that 2*E*(*t*)≤|*K* ^1/2^ *u*′|^2^ + *α* | *A* ^1/2^ *u*|^2^. Thus we deduce that

(72) 2βα∫sTE(t)(p+1)/2dt ≤−[E(t)(p−1)/2(Ku′,u)]sT  +p−12∫sTE(t)(p−3)/2E′(t)(Ku′,u)dt  +∫sTE(t)(p−1)/2((1+α−1)|K1/2u′|2−(g(u′),u))dt ≡I1+I2+I3.

Using the continuity of the imbedding *V* ⊂ *H*, the Cauchy-Schwarz and the Young inequalities, we obtain

(73) $$\begin{array}{c} \left|\left(Ku^{\prime },u\right)\right|\leq c\left|Ku^{\prime }\right|||u||\leq cE\left(t\right). \end{array}$$

Hence, since *E*(*t*) is nonincreasing, we obtain

(74) I1≤cE(p−1)/2(0)E(s),I2≤(p−1)2∫sTcE(t)(p−1)/2E′(t)dt≤cE(p−1)/2(0)E(s).

In order to estimate the last term *I*_3_ of (72), we set

(75) $$\begin{array}{c} \Omega_{1}=\left\{x\in \Omega :\left|K^{1/2}u^{\prime }\left(t,x\right)\right|\leq 1\right\},\\ \Omega_{2}=\left\{x\in \Omega :\left|K^{1/2}u^{\prime }\left(t,x\right)\right|>1\right\}. \end{array}$$

Then we have

(76) ∫Ω|K1/2u′(t,x)|2dx=∫Ω1|K1/2u′(t,x)|2dx+∫Ω2|K1/2u′(t,x)|2dx.

The Hölder inequality yields

(77) ∫Ω|K1/2u′(t,x)|2dx≤c(∫Ω1|K1/2u′(t,x)|p+1dx)2/(p+1)+∫Ω2|K1/2u′(t,x)|2dx≡J1+J2.

Using (65) and (68), we deduce that

(78) J1≤c(∫Ω1u′g(u′)dx)2/(p+1)≤c|E′(t)|2/(p+1),J2≤c∫Ω2|u′g(u′)|dx≤c(−E′(t)).

Combining these two inequalities with (77), we obtain

(79) $$\begin{array}{c} \int_{\Omega }{\left|K^{1/2}u^{\prime }\left(t,x\right)\right|}^{2}dx\leq c{\left(-E^{\prime }\left(t\right)\right)}^{2/(p+1)}+c\left(-E^{\prime }\left(t\right)\right). \end{array}$$

Applying Young's inequality, it follows that, for any *ϵ* > 0,

(80) ∫sTE(t)(p−1)/2|K1/2u′|2dt ≤ϵc∫sTE(t)(p+1)/2dt  +c(ϵ(1−p)/2+E(p−1)/2(0))E(s).

It remains to estimate the second term of *I*_3_. Using (88) we have

(81) |∫Ω1ug(u′)dx| ≤c||u||L(p+1)/p(Ω1)||g(u′)||Lp+1(Ω1) ≤c||u||L(p+1)/p(Ω1)(∫Ω1u′g(u′)dx)1/(p+1) ≤cE(t)1/2    (−E′(t))1/(p+1).

Similarly, using (6), we obtain

(82) |∫Ω2ug(u′)dx|≤c||u||L2(Ω2)||g(u′)||L2(Ω2)≤c||u||L2(Ω2)||u′g(u′)||L1(Ω2)1/2≤cE(t)1/2(−E′(t))1/2.

From (81) and (82), we deduce

(83) |∫Ωug(u′)dx|≤cE(t)1/2(−E′(t))1/(p+1)+cE(t)1/2(−E′(t))1/2.

Using Young's inequality and

(84) $$\begin{array}{c} E{\left(t\right)}^{p/2}{\left(-E^{\prime }\left(t\right)\right)}^{1/2}=E{\left(t\right)}^{(p+1)/4}\left(E{\left(t\right)}^{(p-1)/4}{\left(-E^{\prime }\left(t\right)\right)}^{1/2}\right), \end{array}$$

it follows from (82) that, for any *ϵ* > 0,

(85) −∫sTE(t)(p−1)/2(g(u′),u)dt =−∫sTE(t)(p−1)/2∫Ωug(u′)dx dt ≤c∫sTE(t)p/2(−E′(t))1/(p+1)dt  +c∫sTE(t)p/2(−E′(t))1/2dt ≤2ϵc∫sTE(t)(p+1)/2dt  +c(ϵ−p+ϵ−1E(0)(p−1)/2)  ×∫sT(−E′(t))dt ≤2ϵc∫sTE(t)(p+1)/2dt  +c(ϵ−p+ϵ−1E(0)(p−1)/2)E(s).

Combining (80) with (85) and setting $\tilde{\alpha }=1+\alpha^{-1}$, we obtain

(86) I3≤∫sTE(t)(p−1)/22|K1/2u′|2dt+∫sTE(t)(p−1)/2∫Ωug(u′)dx dt≤(α~+2)ϵc∫sTE(t)(p+1)/2dt+c(α~ϵ(1−p)/2+ϵ−p  +(α~+ϵ−1)E(0)(p−1)/2)E(s).

Therefore we conclude that

(87) (2βα−(α~+2)ϵc)∫sTE(p+1)/2 ≤c(α~ϵ(1−p)/2+ϵ−p   +(α~+ϵ−1)E(0)(p−1)/2)E(s).

Now we choose *ϵ* as *ϵ* ∈ (0,2*β*/(3*α* + 1)*c*); then (69) follows.

## 4. Numerical Result

In this section, we consider a Kirchhoff-type equation with heterogeneous string as an application:

(88) (A(x)ρ)u′′(x,t)−(1+∫01|∇u(x,t)|2dx)Δu(x,t) +κ|u′(x,t)|2u′(x,t)=0,

(89) $$\begin{array}{c} \text{in}\;\;\left(x,t\right)\in \left(0,1\right)\times \left(0,3\right), \end{array}$$

(90) $$\begin{array}{c} u\left(0,t\right)=u\left(1,t\right)=0\;\mathrm{on}\;\left(0,3\right), \end{array}$$

(91) $$\begin{array}{c} u_{0}=u\left(x,0\right)=\exp\left(-64{\left(x-\frac{1}{2}\right)}^{2}\right)\;\text{in}\;\left(0,1\right), \end{array}$$

(92) $$\begin{array}{c} u_{1}=u_{t}\left(x,0\right)=0\;\text{in}\;\left(0,1\right), \end{array}$$

where *κ* is a positive constant and *A*(*x*), *ρ* are given in Table 1.

Then, the operators *K* = *A*(*x*)*ρI*(*I* : *H* → *H*; identity  operator), *A* = −Δ, and the functions *M*(*s*) = *s* + 1 and *g*(*x*) = *κ* | *x*|^2^ *x* so that we can easily check that the hypotheses (M), (G), (H), and (S) in Preliminaries are satisfied. The smallness condition satisfies (||∇*u*_0_||^2^ + 1)||Δ*u*_0_||^2^ ≈ 0.213 ≤ 1/4. Therefore, by Theorem 1, we can deduce the following results.

**Theorem 4**

For any *T* > 0, there is a unique solution *u* ∈ *L* ^*∞*^(0, *T*; *H* ^2^(0,1))  ∩  *W* ^1,*∞*^(0, *T*; *H*_0_^1^(0,1))∩*W* ^2,*∞*^(0, *T*; *L* ^2^(0,1)) to the system (88)–(92).

The energy for the system (88)–(92) is given by

(93) E(t)=12[∫01|A(x)ρu′(x,t)|2dx+∫01|∇u(x,t)|2dx+12(∫01|∇u(x,t)|2dx)2].

Next, in order to get the energy decay of (88)–(92), we need the value of the parameter *p* in (65). We can easily check that *p* = 3 when *g*(*x*) = *κ* | *x*|^2^ *x*.

Therefore, by Theorem 3, we get the energy decay rates for the energy *E*(*t*) as follows.

**Theorem 5**

We obtain the following energy decay:

(94) $$\begin{array}{c} E\left(t\right)\leq c_{1}{\left(1+t\right)}^{-1}\;\forall t\geq 0, \end{array}$$

where *c*_1_ is a positive constant.

For the numerical simulation, we use the finite difference methods (FDM) which are the implicit multistep methods in time and second-order central difference methods for the space derivative in space in numerical algorithms (see [8, 9, 11]).

**Figure 1:**
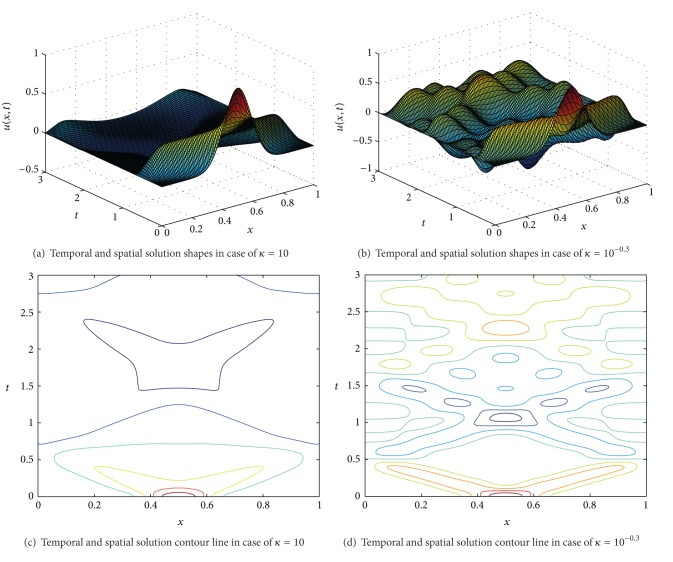
Solution shapes and contour lines with respect to *κ* = 10 and *κ* = 10^−0.3^.

Figures 1(a)–1(d) show displacements of solutions to the system (88)–(92) with *κ* = 10 and *κ* = 10^−0.3^, respectively.

**Figure 2:**
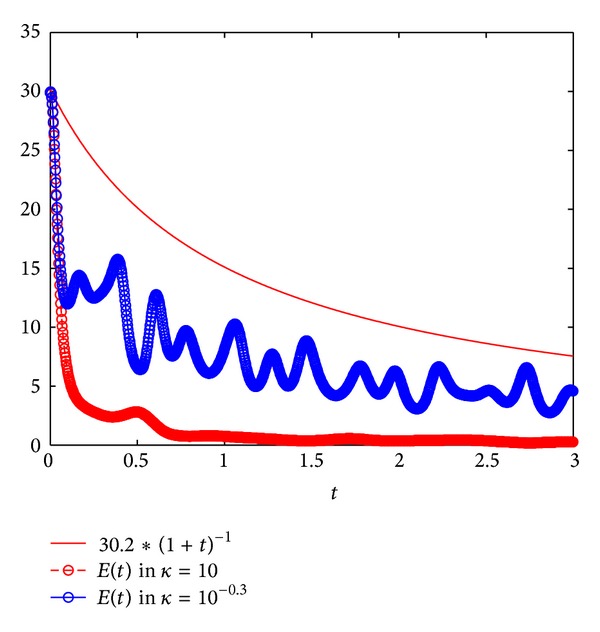
Algebraic decay rates of the energy in case of *κ* = 10 and *κ* = 10^−0.3^.

In case of *κ* = 10 and *κ* = 10^−0.3^, we deduce the algebraic decay rate for the energy as shown in Figure 2, respectively. The blue line and red dotted circled line (or blue circled line) show *c*_1_(*t* + 1)^1^ and *E*(*t*) per the two values, respectively, where the parameter value *c*_1_ = 30.2 in (94). This result shows that the energy decay rates for solutions are algebraic in case that the system (88)–(92) with the nonlinear damping term *κ*|*u*_*t*_|^2^ *u*_*t*_.

## Figures and Tables

**Table 1:** Simulation parameters which are satisfied by theoretical conditions.

Symbols	Definition	Values	Reference
*A*(*x*)	Cross-sectional area	0.7853(10^−4^sin⁡⁡(2^10^ *πx*) + 1) cm^2^	[34]
*ρ*	Mass density of the unit length	7.850 g/cm^2^	[34]
